# Antibacterial Effects of Sodium Borate and Calcium Borate Based Polymeric Coatings for Orthopedic Implants

**DOI:** 10.7759/cureus.22173

**Published:** 2022-02-13

**Authors:** Huseyin S Coskun, Lokman Kehribar, Serkan Surucu, Mahmud Aydin, Mahir Mahirogullari

**Affiliations:** 1 Orthopedics and Traumatology, Ondokuz Mayıs University, Samsun, TUR; 2 Orthopedics and Traumatology, Samsun Gazi State Hospital, Istanbul, TUR; 3 Orthopedics, University of Missouri - Kansas City, Kansas City, USA; 4 Orthopedics and Traumatology, Haseki Education and Research Hospital Polyclinics, Istanbul, TUR; 5 Orthopedics and Traumatology, Memorial Sisli Hospital, Istanbul, TUR

**Keywords:** sodium borate, implant surface, calcium borate, biofilm layers, antibacterial

## Abstract

Introduction

Implants used in orthopedic surgery can be colonized by bacteria that form biofilm layers complicating treatment. We aimed to determine titanium implants' antibacterial and biofilm-degrading properties when coated with sodium borate (NaB) and calcium borate (CaB) minerals.

Methods

We analyzed twenty-four different implants. Three implants were not coated, three were coated with only a carrier polymer (alginate), and eighteen were coated with either CaB or NaB at different concentrations. The implants were incubated with *Staphylococcus aureus*, and then the bacterial colonies were enumerated.

Results

The highest microbial load was observed on the implant coated with alginate (1000 colony-forming units [CFU]/mL). The implant without coating contained a microbial load of 420 CFU/mL. The microbial loads of the implants coated with 0.75 mg/mL CaB or 0.25, 0.5, and 0.75 mg/mL NaB (100, 200, 0, and 0 CFU/mL, respectively) were lower than that of the implant without coating. No biofilm formation was observed on implant surfaces coated with 0.5 mg/mL NaB, 0.75 mg/mL NaB, or 0.75 mg/mL CaB; biofilm formation was observed on the implant without coating and alginate-coated implants surfaces.

Conclusion

At high concentrations, borate minerals (NaB and CaB) have a potent antibacterial effect on colonization and biofilm formation on the implant surface. These elements may be used in implant coating in the future because of their potential antibacterial effects.

## Introduction

Various implants are used in orthopedic surgery [1]. Implants used in orthopedic surgery are expected to show high biocompatibility and biomechanical durability [2]. Recent studies have attempted to develop implants with antibacterial properties in addition to these characteristics [3, 4]. The colonization of microorganisms and especially the formation of a biofilm layer around the implant create complications that impact treatment [5]. Biofilm layers comprise polysaccharides that protect bacteria from antibiotics and host defenses. Once a biofilm layer has formed, the treatment of infection becomes complicated. Following the formation of a biofilm layer, microorganisms enter a stable growth phase in which their metabolic activities decrease, and they become resistant to antimicrobial agents [6]. The biofilm layer is resistant to various antibiotics; however, various molecules that affect the biofilm layer have been reported recently [7]. Implant coating for the prevention of biofilm is not a new subject. In recent literature, many well-defined elements demonstrated antibacterial activity against the biofilm. The most well-known of these is silver [8].

Borate minerals are increasing interest for their notable biological performance, e.g., in contact with bone and soft tissues [9, 10]. Wound healing effects of borate minerals are well defined in the literature [11]. Borate compounds also exhibit potent antibacterial activities, and their use in various fields has increased in recent years [12]. All types of borate minerals are found to be "bioactive" based on two mechanisms: (i) the formation of an apatite layer on the surface during the dissolution of the bioactive glass in a physiological environment, and (ii) the release of biologically active ions [13].

This study focused on exploring the antibacterial applications of borate and nobleite minerals, which comprise borate oxides of sodium and calcium. We aimed to investigate the biofilm-degrading and antibacterial properties of calcium borate (CaB) and sodium borate (NaB) coatings for titanium screws, one of the most frequently used implants in orthopedic surgery.

## Materials and methods

Implant surface modification

Three screws were coated with 0.25 mg/mL calcium borate (CaB25), three screws with 0.50 mg/mL calcium borate (CaB50) and three screws with 0.75 mg/mL calcium borate (CaB75), respectively. Three screws were coated with 0.25 mg/mL sodium borate (NaB25), three screws with 0.50 mg/mL sodium borate (NaB50) and three screws with 0.75 mg/mL sodium borate (NaB75), respectively.

Alginate (ALG) is a natural polysaccharide that is easily modified based on the sugar groups present in its structure. It is widely used in the development of biomaterials [14-16]. Since the implants can not be coated with the aforementioned molecules in isolation, ALG has been used as a carrier polysaccharide in our study. In order to better distinguish whether the anti-bacterial effect is due to the alginate or the coated molecule, an additional isolated alginate group with three screws was created. Three screws were not coated with any material and were used for the control group (Table 1). Data is presented as the average within a 95% confidence interval. The standard deviation was 5% for each set.

**Table 1:** Implants treated with borate minerals at different concentrations. CaB: Calcium borate, NaB: Sodium borate, ALG: Alginate

Implant	CaB, mg/100 mL	NaB, mg/100 mL	Alginate, 20 mL, 2% v/w
NaB25	-	25	+
NaB50	-	50	+
NaB75	-	75	+
CaB25	25	-	+
CaB50	50	-	+
CaB75	75	-	+
ALG	-	-	+
Plain	-	-	-

Coating process

The polymer coating was developed as follows: 2% (weight/weight [w/v]) stock ALG solution was prepared by dissolving 4 g of an ALG sample in 200 mL of deionized water. The sample was homogenized at room temperature (25-28 °C) to completely dissolve the polymer using a magnetic stirrer (125 rpm) for 24 hours. To prepare solutions containing different borate minerals (CaB, NaB, or ALG), 20 mL of the ALG solution was added to 50 mL falcon tubes. Later, the samples were homogenized again by adding different amounts of CaB and NaB to form small-particle polymer solutions. The titanium implants were immersed in the relevant solutions for 30 minutes. Surface-coated implants were immersed in a CaCl2 solution (20% w/v) for five minutes to enable crosslinking of the carrier polymer. Finally, the alginate crosslinked implants were immersed in alcohol (ethanol, ultrapure) for 15 minutes to remove water from the gel structure. The modified implants were dried in an oven at 37 °C and stored in a refrigerator at 4 °C until use.

Biofilm formation and bacterial culture

*Staphylococcus aureus* ATCC 291213, which produces strong biofilms, was used in the study. The strain was cultured in Luria-Bertani (LB; tryptone 1.0%, yeast extract 0.5%, NaCl 1.0%, agar 2.0%) medium at 37 °C for 24 hours. Subsequently, a turbid bacterial suspension equivalent to 0.5 McFarland standard (approximately 10 colony-forming units [CFU]/mL) was prepared in LB medium supplemented with 0.5% glucose. The implant samples were separately immersed in cell suspensions and placed in a shaking incubator at 37 °C for 48 hours to investigate biofilm formation [17]. 

Culture analysis

The implant samples were placed into sterile falcon tubes containing 10 mL of sterile saline and vortexed for three minutes. The bacterial biofilm layer on the surface of each sample was removed and homogenized to form a bacterial suspension. To enumerate bacterial colonies, a series of dilutions were prepared from the bacterial suspensions obtained from each sample. Aliquots (100 µL) of each dilution were inoculated into LB agar plates, which were incubated in a static oven at 37 °C for 48 hours. The resulting bacterial colonies were counted, and the results were expressed in CFU/mL.

Scanning electron microscopy analysis

The biofilm-containing implant surfaces were analyzed via scanning electron microscopy (SEM) to observe changes in surface morphology. Scanning electron microscope (SEM) images were obtained with a JSM-7001F STEM (JEOL Ltd., Tokyo, Japan). The examination was performed under 10.000x magnification and analyzed by a member of the histology department. Morphologic changes on the surface of each sample have been recorded. 

## Results

Twenty-four implants were analyzed. The microbial load of the control group implant surface was 420 CFU/mL. The microbial load of the ALG implant (1000 CFU/mL) was 2.4-fold higher than that of the control group implant. The antibacterial activity of implants modified with CaB increased with increased mineral concentration. The CaB25 implant, coated with 0.25 mg/mL CaB, exhibited an increase in antibacterial activity of 2% and a lower microbial load (980 CFU/mL) relative to the control group implant. When the CaB concentration was increased to 0.5 and 0.75 mg/mL, the antibacterial activity increased by up to 24% and 90%, respectively. The microbial load decreased to 760 and 100 CFU/mL, respectively, compared to the control group implant. 

In implants coated with the lowest concentration of NaB (25 mg/mL), an increase in antibacterial activity of 80% and a microbial load of 200 CFU/mL were observed compared to the control group. No colony formation occurred at increased concentrations of NaB (0.5 and 0.75 mg/mL).

**Figure 1:**
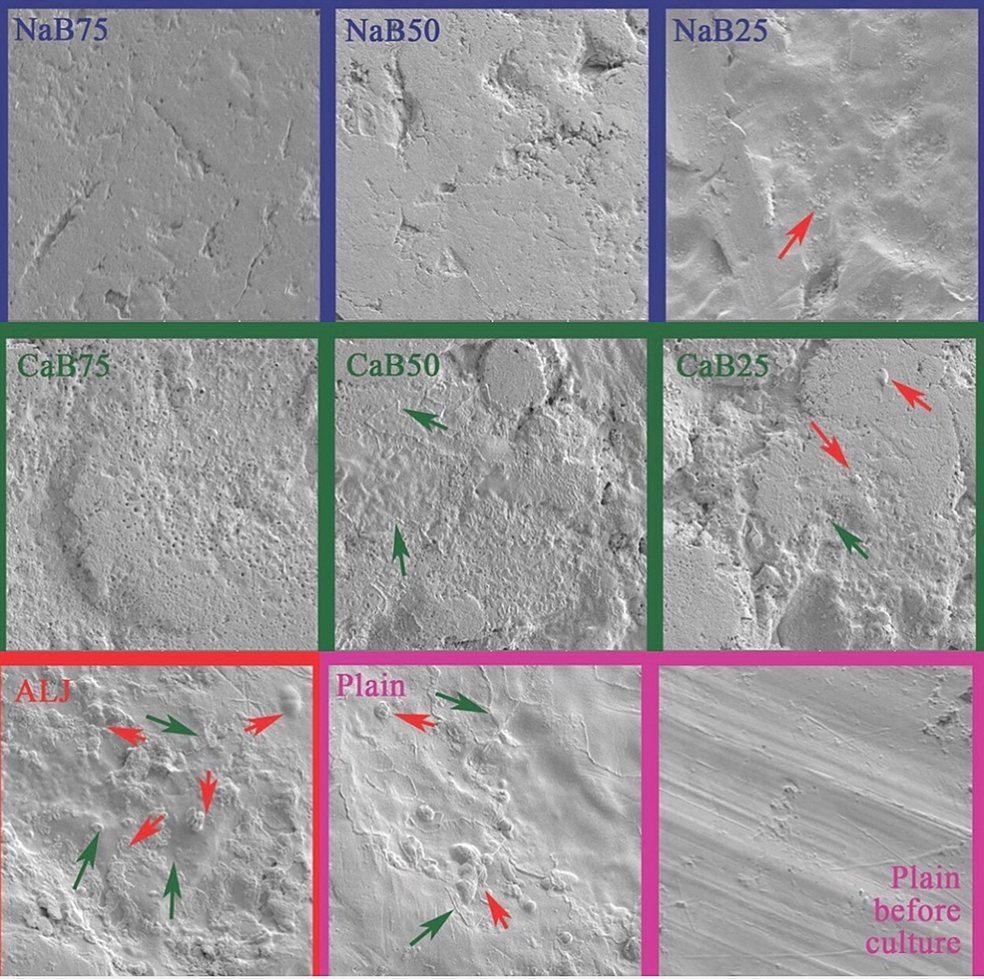
The scanning electron microscope images Green arrows indicate the "clear" zone around the bacterial colonization.  In CaB 50 group, since no bacterial colonization was observed, this green arrow is indicating the rough zone around the biofilm formation. Red arrows indicate bacterial colonization areas.

The scanning electron microscope images indicated that the implant surface became relatively rougher after applying the coatings (Figure 1). A higher degree of bacterial colonization and biofilm formation was observed on ALG implant surfaces than on control group implant surfaces (Figure 1). While bacterial colonization and biofilm formation were observed in CaB25, biofilm formation alone was observed in CaB50. No bacterial colonization or biofilm residues were observed in the scanned area of ​​implants coated with 0.75 mg/mL CaB. 

The bacterial biofilm layers were investigated after examining the implant surfaces via electron microscopy. No biofilm formation or bacterial colony growth was observed on the NaB75, NaB50, or CaB75 implants. Biofilm formation and bacterial colony growth were observed on the surfaces of the other groups, most prominently on the ALG group.

## Discussion

Surgical site infections in orthopedic surgery are a challenge for both physicians and patients. Once osteomyelitis occurs, the treatment becomes even harder, including the risk of losing extremity or even death. Therefore, implant coating with anti-bacterial molecules is getting more interested in the literature. In accordance with this purpose, bioactive glasses have been used as coatings for biomedical implants because they can be formulated to promote osseointegration, anti-bacterial behavior, bone formation, and tissue healing through the incorporation and subsequent release of certain ions. In a study examining the mechanical effects of borate minerals, results are found close to titanium alloys [18].

In another study, the ability of silver (Ag)-containing bioactive borate glass (BG) coatings to improve the biocompatibility and anti-bacterial properties of titanium (Ti) implants was investigated in vitro and in vivo in a rabbit tibial fracture model. The authors compared different coatings of bioactive borate glasses, 0, 0.75, and 1.0 mass percentage (wt%) Ag20. Borate BG coatings (;20-lm thick) containing 0.75 or 1.0 wt% Ag2O improved the biocompatibility and anti-bacterial properties of Ti implants. Titanium implants coated with BG containing 1.0 wt% Ag2O (designated BG-1.0Ag) were most effective for simultaneously supporting fracture fixation and eradicating methicillin-resistant *Staphylococcus aureus* (MRSA)-induced infection within six weeks in a rabbit tibial model. The authors concluded that implants coated with Ag-containing borate coatings could provide an approach for reducing implant-related bone infection [19]. The borate coating amounts in our study were planned with inspiration from this study. 

To the best of our knowledge, no anti-bacterial studies regarding sodium borate were found in the literature. In a study, authors investigated the transformation of two sodium calcium borate glasses to hydroxyapatite [20]. This can be considered in fracture healing studies thanks to its transformation properties. 

Bacterial contamination in orthopedic surgery is a serious complication that requires prolonged systemic antibiotic therapy and major surgery and is associated with deformity, significant morbidity, and hospital costs. Current treatments based on local delivery of high doses of antibiotics by carrier materials such as polymethyl methacrylate (PMMA) cement (the gold standard for treatment), collagen sponge (fleece), and calcium sulfate are effective, but they suffer from limitations. There is interest in alternative carrier systems that are not only effective in treating implant-related bone infection but are also biodegradable (or absorbable), bioactive, and have the capacity to stimulate bone regeneration [21]. Alginate is used as a carrier polymer in this study, but its effect on cytotoxicity should be carefully investigated before using it in in-vivo models. Bone stimulating effects of CaB and NaB would be a subject of another study to enhance their use in orthopedic surgery. 

Although the insignificant improvement over CaSO4 in eradicating osteomyelitis in a rabbit tibial model [22], the chitosan-bonded borate glass composites showed a better capacity than CaSO4 to promote new bone formation and reconstituting the defect [23]. We noticed very few studies on calcium borate and its effect on fracture healing and bone-defect filling in the current literature. 

On the other hand, several boron-derived materials have been used as coatings and additives for different applications [24]. Various studies have investigated the anti-bacterial properties of boric acid [25]. Our study used two other borate minerals (NaB and CaB) to modify implant surfaces and analyzed their anti-bacterial activity based on biofilm degradation and colony counts. 

While bacterial colonization and biofilm formation were observed in CaB25, biofilm formation alone was observed in CaB50. This indicated that the binding of bacteria to the surface might have decreased as the mineral concentration increased, inhibiting biofilm formation. No bacterial colonization or biofilm residues were observed in the scanned area of ​​implants coated with 0.75 mg/mL CaB. This indicated that this concentration of CaB conferred a potent anti-bacterial effect. The increase in anti-bacterial activity as the dose increases is compatible with the literature, but the cytotoxicity that occurs with increasing dose must be well clarified to prevent complications.

While a small degree of bacterial colonization was observed on the surfaces with the lowest concentration of NaB coating, no bacterial colonization or biofilm formation was observed on surfaces coated with higher concentrations of NaB. This suggested that NaB endows implant surfaces with potent anti-bacterial properties. Although ALG, which was used as a carrier polymer, was shown to negatively affect the anti-bacterial properties of a surface, the addition of borate mineral appeared to inhibit this effect and impart bacterial resistance. 

An interesting detail of the study was observed in the 0.50 mg/mL CaB group. Biofilm formation was observed at this concentration, but no bacterial colonization was observed. This result demonstrates that this concentration is enough to eradicate bacterial colonization but not sufficient for preventing biofilm formation. This concentration of calcium borate can be considered in future in-vivo studies, considering biofilm formation.

The study's limitations included a lack of support from experimental animal studies and the use of only a single bacterial strain to evaluate anti-bacterial activity. Another limitation is the lack of the assessment of the cytotoxicity of the elements mentioned above. Also, the number of implants used in the study could have been higher. 

Finally, the carrier polysaccharide alginate contained more colonies than the control group, which questions the antigenicity of this polysaccharide. This study may be done with other polysaccharides with a lower bacterial ingrowth rate.

## Conclusions

We believe that coating orthopedic implants can prevent bacterial colonization and biofilm formation on the implant surface with borate minerals (NaB and CaB) at high concentrations. Especially NaB coating seems much more effective than the CaB coating. NaB provides an excellent anti-bacterial effect against *Staphylococcus aureus* even in the lower coating levels. This study does not investigate the cytotoxic effects of these molecules. Therefore this subject needs further in-vitro studies with larger samples and different pathogens before using the coating in in-vivo conditions.
